# The rs11684747 and rs55790676 SNPs of ADAM17 influence tuberculosis susceptibility and plasma levels of TNF, TNFR1, and TNFR2

**DOI:** 10.3389/fmicb.2024.1392782

**Published:** 2024-05-31

**Authors:** José Alberto Choreño-Parra, Lucero A. Ramon-Luing, Manuel Castillejos, Emmanuel Ortega-Martínez, Alan Rodrigo Tapia-García, Melvin Barish Matías-Martínez, Alfredo Cruz-Lagunas, Gustavo Ramírez-Martínez, Itzel Alejandra Gómez-García, Jazmín Ariadna Ramírez-Noyola, Beatriz Garcia-Padrón, Karen Gabriel López-Salinas, Fabiola Jiménez-Juárez, Parménides Guadarrama-Ortiz, Citlaltepetl Salinas-Lara, Karolina Bozena-Piekarska, Marcela Muñóz-Torrico, Leslie Chávez-Galán, Joaquín Zúñiga

**Affiliations:** ^1^Dirección de Enseñanza, Instituto Nacional de Enfermedades Respiratorias Ismael Cosio Villegas, Mexico City, Mexico; ^2^Laboratory of Immunobiology and Genetics, Instituto Nacional de Enfermedades Respiratorias Ismael Cosío Villegas, Mexico City, Mexico; ^3^Laboratory of Integrative Immunology, Instituto Nacional de Enfermedades Respiratorias Ismael Cosío Villegas, Mexico City, Mexico; ^4^Departamento de Epidemiología Hospitalaria e Infectología, Instituto Nacional de Enfermedades Respiratorias Ismael Cosío Villegas, Mexico City, Mexico; ^5^Posgrado en Ciencias Quimicobiológicas, SEPI, Escuela Nacional de Ciencias Biológicas, Instituto Politécnico Nacional, Mexico City, Mexico; ^6^Department of Pathology, Instituto Nacional de Neurología y Neurocirugía Manuel Velasco Suárez, Mexico City, Mexico; ^7^Red MEDICI, Facultad de Estudios Superiores Iztacala, Universidad Nacional Autónoma de México, Tlalnepantla de Baz, Mexico; ^8^Tecnologico de Monterrey, Escuela de Medicina y Ciencias de la Salud, Mexico City, Mexico; ^9^Sección de Posgrado e Investigación, Escuela Superior de Medicina, Instituto Politécnico Nacional, Mexico City, Mexico; ^10^Centro Especializado en Neurocirugía y Neurociencias México, Mexico City, Mexico; ^11^Clínica de Tuberculosis, Instituto Nacional de Enfermedades Respiratorias Ismael Cosío Villegas, Mexico City, Mexico

**Keywords:** tuberculosis, ADAM17, TNF-α converting enzyme, tumor necrosis factor, tumor necrosis factor receptors

## Abstract

**Introduction:**

The proteolytic activity of A Disintegrin and Metalloproteinase 17 (ADAM17) regulates the release of tumor necrosis factor (TNF) and TNF receptors (TNFRs) from cell surfaces. These molecules play important roles in tuberculosis (TB) shaping innate immune reactions and granuloma formation.

**Methods:**

Here, we investigated whether single nucleotide polymorphisms (SNPs) of ADAM17 influence TNF and TNFRs levels in 224 patients with active TB (ATB) and 118 healthy close contacts. Also, we looked for significant associations between SNPs of ADAM17 and ATB status. TNF, TNFR1, and TNFR2 levels were measured in plasma samples by ELISA. Four SNPs of ADAM17 (rs12692386, rs1524668, rs11684747, and rs55790676) were analyzed in DNA isolated from peripheral blood leucocytes. The association between ATB status, genotype, and cytokines was analyzed by multiple regression models.

**Results:**

Our results showed a higher frequency of rs11684747 and rs55790676 in close contacts than ATB patients. Coincidentally, heterozygous to these SNPs of ADAM17 showed higher plasma levels of TNF compared to homozygous to their respective ancestral alleles. Strikingly, the levels of TNF and TNFRs distinguished participant groups, with ATB patients displaying lower TNF and higher TNFR1/TNFR2 levels compared to their close contacts.

**Conclusion:**

These findings suggest a role for SNPs of ADAM17 in genetic susceptibility to ATB.

## 1 Introduction

Tuberculosis (TB), an infectious disease caused by *Mycobacterium tuberculosis* (Mtb), is a global public health problem which caused 7.5 million new cases and 1.3 million deaths in 2022 (Global Tuberculosis Report 2023, n.d.). Although a primary infection can induce the active form of TB (ATB), most ATB cases derive from latent tuberculosis (LTB) progression, a status of asymptomatic infection without microbiological evidence of bacterial viability but positive readouts of cellular immune memory against Mtb (Tufariello et al., 2003; Mack et al., 2009; Getahun et al., 2015). The last estimation of LTB burden indicated that approximately 1.7 billion individuals were latently infected with Mtb in 2014, representing about a quarter of the global population (Houben and Dodd, 2016). From these, only 5 to 10% will progress to ATB each year with the lung as the main organ affected. Currently, the factors associated with progression to ATB are not completely understood despite its epidemiological relevance. Of course, several genetic, demographic, sociocultural, microbiological, and nutritional characteristics, along with comorbid conditions of cases may affect protective anti-TB immunity. Nonetheless, given the complexity of host-pathogen interactions in TB, a sole immune parameter determinant of protection or pathology has not been found.

For many years, the paradigm of TB immunopathogenesis has established that Mtb infection control greatly relies on memory immune responses of CD4+ T helper cells, which produce interferon-gamma (IFN-γ) to assist macrophages in exploiting their full bactericidal capacity (Shimokata et al., 1986; Flynn et al., 1993; Orme et al., 1993). Unfortunately, the vaccine candidates based on this dogma have failed in providing protection in the real world (Tameris et al., 2013), and IFN-γ-release assays (IGRAs) have no prognostic value to predict ATB progression in individuals with LTB (Diel et al., 2012; Aboudounya and Heads, 2021). Thus, a higher interest in searching for other protective mechanisms has led to striking findings that link previously unrecognized roles of innate immunity and TB protection operating in LTB but not ATB individuals (Khader et al., 2019; Esaulova et al., 2021).

The innate phase of the anti-TB immune response is dominated by alveolar macrophages and recruited monocytes, which participate in granuloma formation, thereby impeding Mtb dissemination (Tsai et al., 2006; Dunlap et al., 2018). Tumor necrosis factor (TNF), a proinflammatory cytokine of innate immunity, plays an essential role in the formation and maintenance of granulomas, macrophage recruitment and nitric oxide production, and the development of giant multinucleated cells (Kindler et al., 1989; Flynn et al., 1995; Roach et al., 2002; Mezouar et al., 2019). Thus, TNF is pivotal in mediating immune protection against Mtb during the early and late stages of infection, as demonstrated by the increased susceptibility to ATB progression among people with LTB receiving anti-TNF immunotherapies (Keane et al., 2001; Gómez-Reino et al., 2003; Mohan et al., 2004; Wallis et al., 2004). Also, diverse reports indicate that single-nucleotide polymorphisms (SNPs) increase the risk of developing TB by decreasing TNF levels or interfering with its signaling (Wu et al., 2019; Adane et al., 2021; Souza de Lima et al., 2021).

TNF is produced by several leucocyte subsets (Dorhoi and Kaufmann, 2014), and it is expressed as a trimeric transmembrane protein (tmTNF) or a soluble form (solTNF). Both TNF forms are bioactive when they attach to TNF receptor 1 (TNFR1), which mediates inflammation and tissue injury, or TNF receptor 2 (TNFR2), which attenuates excessive inflammation during mycobacterial infection (Chavez-Galan et al., 2017, 2019; Ruiz et al., 2021). TNFRs are also expressed on the cell surface as trans-membrane proteins (tmTNFR1 and tmTNFR2) or released from the cell as soluble forms (solTNFR1 and solTNFR2). Whereas tmTNFR1 and tmTNFR2 induce intracellular signaling after the binding with tmTNF or solTNF (Ruiz et al., 2021), soluble TNFRs are anti-inflammatory mediators acting as decoy receptors to neutralize TNF (Kohno et al., 1990). The shedding of TNF and its receptors from the cell membrane is mediated by the proteolytic activity of A Disintegrin and Metalloproteinase 17 (ADAM17), also known as TNF converting enzyme (TACE) (Black et al., 1997; Mohan et al., 2002). Of note, despite the relevance of ADAM17 in regulating TNF and TNFRs levels in diverse diseases (Moss and Minond, 2017; Zunke and Rose-John, 2017), the role of this protease in TB susceptibility has not been assessed.

SNPs of ADAM17 have been implicated in several disorders, including Parkinson’s disease, allergy, sepsis, and vascular diseases (Li et al., 2014, 2019; Shao et al., 2016; He et al., 2022; Jung and Hwang, 2022). Hence, this study aimed to characterize the prevalence of SNPs of ADAM17 among people with ATB and their healthy close contacts and the effect of these polymorphisms in the levels of soluble TNF, TNFR1, and TNFR2. Our findings indicate that people with the rs11684747 and rs55790676 SNPs of ADAM17 display higher plasma levels of solTNF but lower concentrations of TNFRs. Remarkably, the frequency of these SNPs of ADAM17 was higher among people without clinical manifestations of the disease despite being in close contact with ATB cases, especially in those with LTB, as defined by positive IGRA results. Finally, logistic regression analyses showed that homozygosity to the ancestral alleles of both variants is associated with a higher risk for ATB as compared to carriers of rs11684747 and rs55790676 SNPs of ADAM17. Our study elucidates a possible role of ADAM17 in immunity against Mtb and disease susceptibility that deserves further research.

## 2 Materials and methods

### 2.1 Study population

Peripheral blood samples were obtained from ATB patients and their household contacts. All participants were enrolled at the TB clinic of the Instituto Nacional de Enfermedades Respiratorias Ismael Cosío Villegas (INER) in Mexico City. The diagnosis of ATB was laboratory-confirmed by positive results in sputum smear microscopy, sputum/bronchoalveolar lavage (BAL) culture, and GeneXpert MTB/RIF test (Cepheid, CA, United States). The LTB group included household contacts, who shared the same enclosed living space for one or more nights per week or extended periods during the day with TB patients three months before enrollment. All these contacts did not show ATB symptoms, and some of them had a positive result in the IGRA QuantiFERON^®^-TB Gold Plus test (QIAGEN, Hilden, Germany; hereinafter named QTF+). QTF+ participants were considered as having LTB infection and were subjected to clinical evaluation and chest X-ray. The individuals with QTF− were considered as healthy household contacts. QTF+ and QTF− individuals were analyzed as a single group called “No ATB” because they displayed the same behavior in statistical analyses (data indicated in Supplementary material).

Solid-organ transplant recipients and patients with human immunodeficiency virus infection, receiving immunosuppressive treatment, and diagnosed with cancer, or autoimmune diseases were excluded from the study. Clinical and demographic data from participants were obtained by direct clinical interview, physical examination, and review of their medical records.

### 2.2 Samples

Peripheral blood samples were obtained into BD Vacutainer^®^ EDTA tubes as soon as the diagnosis was made and before treatment initiation for ATB patients, or upon acceptance to participate in the study by healthy-close contacts. Peripheral blood mononuclear cells (PBMCs) were isolated by centrifugation gradient using Ficoll-Paque^™^ PLUS (GE Healthcare, Life Sciences, PA, United States) for DNA isolation. Plasma aliquots for protein determinations were stored at −70°C until use.

### 2.3 DNA isolation and genotyping

Genomic DNA was extracted from PBMCs using silica columns (DNeasy^®^ Blood & Tissue Kit, Qiangen^™^, MD, United States) following the manufacturer’s guidelines. Concentration and purity of DNA obtained were determined by measuring the absorbance within the micro-volume spectrophotometer NanoDrop^®^ ND-2000c (ThermoFisher Scientific, Waltham, MA, United States).

For genotyping analysis, the selection of SNPs of the ADAM17 gene was based on a previous study that reported the allele frequency of five SNPs analysis in patients with distinct severity of sepsis (Shao et al., 2016), an infectious disorder where control of inflammation is essential in determining the outcome of affected people, as in TB. Herein, four of these five SNPs of ADAM17 were analyzed using specific TaqMan^®^ genotyping assays commercially available (ThermoFisher Scientific, Waltham, MA, United States; rs12692386, assay number C__31588436_10; rs1524668, assay number C__8348383_30; rs11684747, assay number C__1829895_10; rs55790676, assay number C__25942661_10). The RT-PCR reaction was set up in 96-well plates with a mix of 6 μL TaqMan^™^ Universal PCR Master Mix (ThermoFisher Scientific, Waltham, MA, United States), 2 μL of free nuclease water, and 3 μL of DNA samples at 5 ng/μL or free nuclease water for negative controls. Thermal cycling was performed at 55°C for 10 min for reverse transcription, followed by 95°C for 10 min, and then 40 cycles of 60°C for 60 s, 60°C for 30 s using a StepOnePlus^™^ thermocycler (Applied Biosystems, Foster City, CA, United States).

### 2.4 Cytokine levels

Soluble levels of TNF, TNFR1, TNFR2, and TIM-3 were quantified by Enzyme-linked immunosorbent assay (ELISA) following the manufacturer’s protocols (ELISA MAX^™^ Deluxe Set Human TNF-α, BioLegend, CA, USA; and Human TNFRI/TNFRSF1A, TNFRII/TNFRSF1B Immunoassay Quantikine^™^, Human TIM-3 DuoSet ELISA; R&D Sytems, MN, USA). The optical density was measured using a microplate reader spectrophotometer (Imark, Bio-Rad, Hercules, United States) set to 450 nm. All proteins were quantified by comparison with the corresponding standard curve.

### 2.5 Haplotype block construction

This analysis was carried out using Haploview 4.2 software (Barrett et al., 2005), applying the analysis algorithm presented by Gabriel et al. (2002). Linkage disequilibrium (LD) was presented using D′ value. Haplotype-association analysis was performed with Fisher’s exact test between cases and controls.

### 2.6 Ethics statement

The Institutional Review Board of the Instituto Nacional de Enfermedades Respiratorias Ismael Cosío Villegas approved the study protocol code B04-23, approved in February 2023. All participants or their legal guardians provided written informed consent to participate in the investigation in adherence to the Declaration of Helsinki for Human Research. The study was conducted under the Mexican Constitution law NOM-012-SSA3-2012, which establishes the criteria for executing clinical investigations in humans. All the personal data and clinical information were managed according to local legislation.

### 2.7 Statistical analysis

Descriptive statistics were used to characterize the study cohorts. Frequencies and proportions were calculated for categorical data. Medians, interquartile ranges, and 95% confidence intervals were used for continuous variables. Comparisons between groups were made using a Chi-square test, Fisher’s exact test, Mann–Whitney U test, or Kruskal–Wallis with *post hoc* Dunn’s test, as appropriate.

A multivariate logistic regression analysis was conducted to identify associations between TB status (ATB) and the soluble levels of TNF, TNFR1 and TNFR2, and SNPs of ADAM17. Thus, multiple logistic regression with three-way interactions was performed with variables that showed statistically significant differences between groups; rs11684747 (AA allele) and rs55790676 (GG allele) SNPs of ADAM17 (which were significantly different in ATB versus no ATB) were defined as dependent variables.

All analyses were performed using GraphPad Prism V 9.0.2 (GraphPad Software, Inc., San Diego, CA, United States) and R statistical software version 4.2.1 (R Foundation for Statistical Computing, Vienna, Austria). *p*-values ≦ 0.05 were considered statistically significant.

## 3 Results

### 3.1 Clinical characteristics of participants

This study enrolled a total of 342 participants, 224 with ATB and 118 house close contacts. The second group included 70 individuals with LTB (QTF+) and 48 QTF−. Supplementary Table S1 summarizes the main demographic and clinical characteristics of the study groups. Since QTF+ and QTF− individuals showed similar features, they were consolidated as a single group for some comparisons with ATB patients. Also, given that both groups represent individuals with higher protection against Mtb, analyses of SNPs were made between this consolidated group and ATB. Overall, 60% of participants were women with a median age of 44 years (Table 1). The ATB group significantly differed from close contacts by a higher proportion of males, lower weight, and body mass index (BMI), and higher prevalence of tobacco smoking and diabetes. A similar proportion of ATB and no ATB participants (87 and 89%, respectively) received the Bacillus Calmette-Guerin vaccine (BCG) during childhood.

**Table 1:** Demographic and clinical characteristics of study populations.

Characteristics	Overall *N* = 342	ATB		
		Yes^a^ *N* = 224	No^b^ *N* = 118	*p*-value (a vs b)
Group				
QTF−	48 (14%)	0 (0%)	48 (41%)	NA
QTF+	70 (20%)	0 (0%)	70 (59%)	NA
ATB	224 (65%)	224 (100%)	0 (0%)	NA
Male gender	138 (40%)	108 (48%)	30 (25%)	<0.001
Age	44 (31, 56)	44 (31, 57)	44 (30, 54)	0.4
BMI	23 (20, 27)	21 (19, 24)	28 (25, 32)	<0.001
Obesity	48 (14%)	10 (5%)	38 (32%)	<0.001
Low weight	79 (23%)	77 (34%)	2 (2%)	<0.001
Smoking	85 (36%)	57 (47%)	28 (24%)	<0.001
DM	95 (28%)	84 (38%)	11 (9%)	<0.001
SAH	49 (14%)	35 (16%)	14 (12%)	0.3
BCG	249 (88%)	144 (87%)	105 (89%)	0.7

Data are shown as *n* (%) or median (IQR 25,75). Differences between groups were analyzed using Pearson’s Chi-squared test, Kruskal-Wallis rank sum test, or Fisher’s exact test, as appropriate. ATB, active pulmonary tuberculosis; BCG, Bacillus Calmette-Guerin vaccine; DM, diabetes mellitus; BMI, body mass index; QTF, QuantiFERON test; SAH, systemic arterial hypertension; NA, not applicable; TB, tuberculosis.

### 3.2 SNPs of ADAM17 are differentially distributed according to ATB status

**Figure 1:**
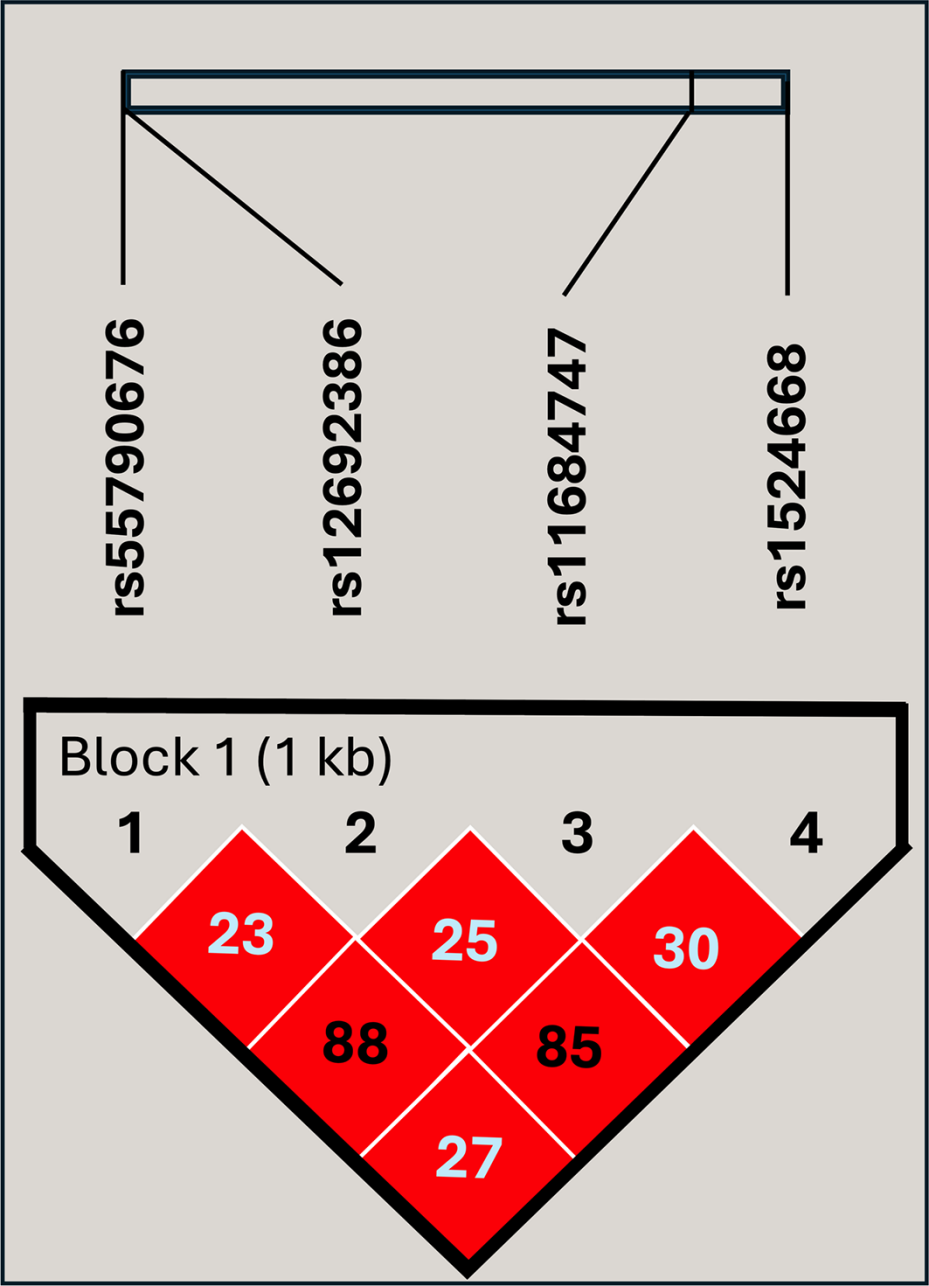
Linkage disequilibrium plot of the genetic variants included in the study. The high intensity of the red diamonds indicates a complete linkage disequilibrium (D′ = 100). The values inside the diamonds are the results of the R^2^ pairwise measures.

To investigate the role of the SNPs of ADAM17 in TB, we analyzed the distribution of rs12692386, rs1524668, rs11684747, and rs55790676 among study groups. First, the possible participation of these SNPs as a cluster of genetic susceptibility was assessed by haplotype blocks. Their genotype distribution among study participants met the Hardy–Weinberg equilibrium (*p* > 0.05) and showed a complete LD (Figure 1). Four blocks of haplotypes were found among cases and controls; however, their frequencies were not different between cases and close contacts (Table 2). Thus, we analyzed each SNP individually.

**Table 2:** Haplotype-association analysis of the evaluated *loci* in the genetic susceptibility to ATB.

Haplotype^a^	ATB		*p*-value^b^
	Yes	No	
GAAA	78	77.6	0.8963
GGAC	13.6	11.6	0.3791
TGGC	5	7.5	0.1395
GGAA	3	2.3	0.5708

a rs55790676-rs12692386-rs11684747-rs1524668.

b Fisher’s exact test. The data are displayed as haplotype frequencies in percentages (%).

The allele frequencies of rs12692386 and rs1524668 were not significantly different between participant groups (Table 3; Supplementary Table S2). In contrast, ATB patients showed a higher frequency of homozygosity to ancestral AA rs11684747 and GG rs55790676 alleles, whereas close contacts were heterozygous AG to rs11684747 and GT to rs55790676 with higher frequency than ATB patients (19% vs. 11%, *p* = 0.027; and 19% vs. 10%, *p* = 0.021, respectively). The allelic frequencies were similar between QTF+ and QTF− close contact groups (Supplementary Table S2). Homozygous to these SNPs of ADAM17 (GG to rs11684747 and TT to rs55790676) were not identified in the cohorts.

**Table 3:** Genotypes and allele frequencies in ATB patients and close contacts.

Characteristics	Overall *N* = 342	ATB		
		Yes^a^ *N* = 224	No^b^ *N* = 118	*p*-value (a vs b)
**rs12692386**				
AA	206 (61%)	138 (62%)	68 (58%)	0.4
AG	112 (33%)	69 (31%)	43 (36%)	0.3
GG	21 (6%)	14 (6%)	7 (6%)	0.9
A	318 (94%)	207 (94%)	111 (94%)	0.9
G	133 (39%)	83 (38%)	50 (42%)	0.4
**rs1524668**				
AA	222 (65%)	151 (68%)	71 (60%)	0.15
AC	100 (29%)	58 (26%)	42 (36%)	0.068
CC	18 (5%)	13 (6%)	5 (4%)	0.5
A	322 (95%)	209 (94%)	113 (96%)	0.5
C	118 (35%)	71 (32%)	47 (40%)	0.15
**rs11684747**				
AA	293 (86%)	198 (89%)	95 (81%)	**0.027**
AG	47 (14%)	24 (11%)	23 (19%)	**0.027**
GG	0 (0%)	0 (0%)	0 (0%)	
A	340 (100%)	222 (100%)	118 (100%)	
G	47 (14%)	24 (11%)	23 (19%)	**0.027**
**rs55790676**				
GG	297 (87%)	201 (90%)	96 (81%)	**0.021**
GT	44 (13%)	22 (9.9%)	22 (19%)	**0.021**
TT	0 (0%)	0 (0%)	0 (0%)	
G	341 (100%)	223 (100%)	118 (100%)	
T	44 (13%)	22 (9.9%)	22 (19%)	**0.021**

Data are displayed as *n* (%). Differences between groups were analyzed using Pearson’s Chi-squared test or Fisher’s exact test, as appropriate. *p* value in bold show a statistically significant difference. a = patients with ATB; b = household contact with no ATB wheter QTF+ or QTF-.

### 3.3 Levels of TNF and TNFRs are influenced by SNPs of ADAM17 and distinguish ATB patients from close contacts

**Figure 2:**
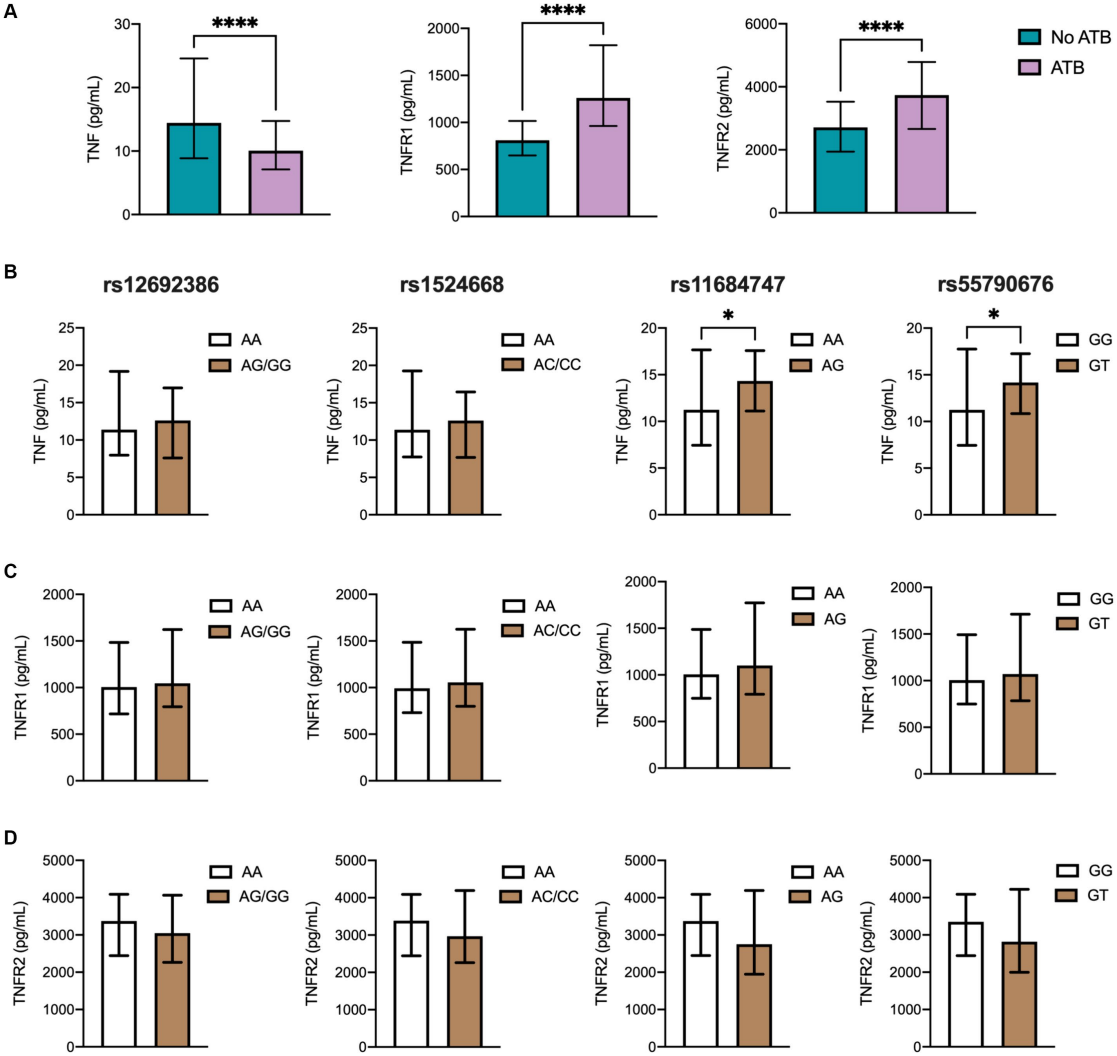
TNF and TNFRs plasma levels in the different TB groups. **(A)** Soluble levels of TNF, TNFR1, and TNFR2 were assessed by ELISA. Genotype distribution according to **(B)** TNF, **(C)** TNFR1, and **(D)** TNFR2 levels in the population, including 224 ATB patients and controls comprehending 70 individuals with LTB (QTF+) and 48 with no LTB (QTF−). Data are displayed as median and IQR values. Differences between groups were performed using the Mann–Whitney U test, *p < 0.05, and **** *p* < 0.0001.

Given the major role of ADAM17 in modulating the release of TNF and TNFRs from cell surfaces, we next investigated an association of SNPs and cytokine levels in TB. These analyses were carried out in the overall study population since all participants were in contact with Mtb, representing biological replicates of the same immunological phenomenon: the production of TNF molecules in response to mycobacteria *in vivo*. First, we measured and compared cytokine levels between study groups, looking for differences that may reflect the distinct grades of protection against Mtb between ATB patients and close contacts. As shown in Figure 2A and Supplementary Table S3, the levels of soluble TNF in plasma were significantly lower in ATB cases than in controls, whereas no differences were observed between QTF+ and QTF− groups (Supplementary Table S4; Supplementary Figure S1). Conversely, TNFR1 and TNFR2 were elevated in ATB patients compared to their close contacts.

Then, a link between SNPs of ADAM17 and TNF, TNFR1, and TNFR2 was evaluated. Notably, homozygous AA to rs11684747 and GG to rs55790676 showed lower plasma levels of TNF than individuals carrying at least one variant allele (Figure 2B). Also, homozygous to ancestral alleles of these SNPs displayed higher levels of TNFR2 that did not reach statistical significance compared to those carrying the variant alleles. No association between rs12692386 and rs1524668 with cytokine levels was found (Figures 2C,D).

### 3.4 Elevated TIM-3 levels in ATB patients and their association with the rs12692386 SNP of ADAM17

**Figure 3:**
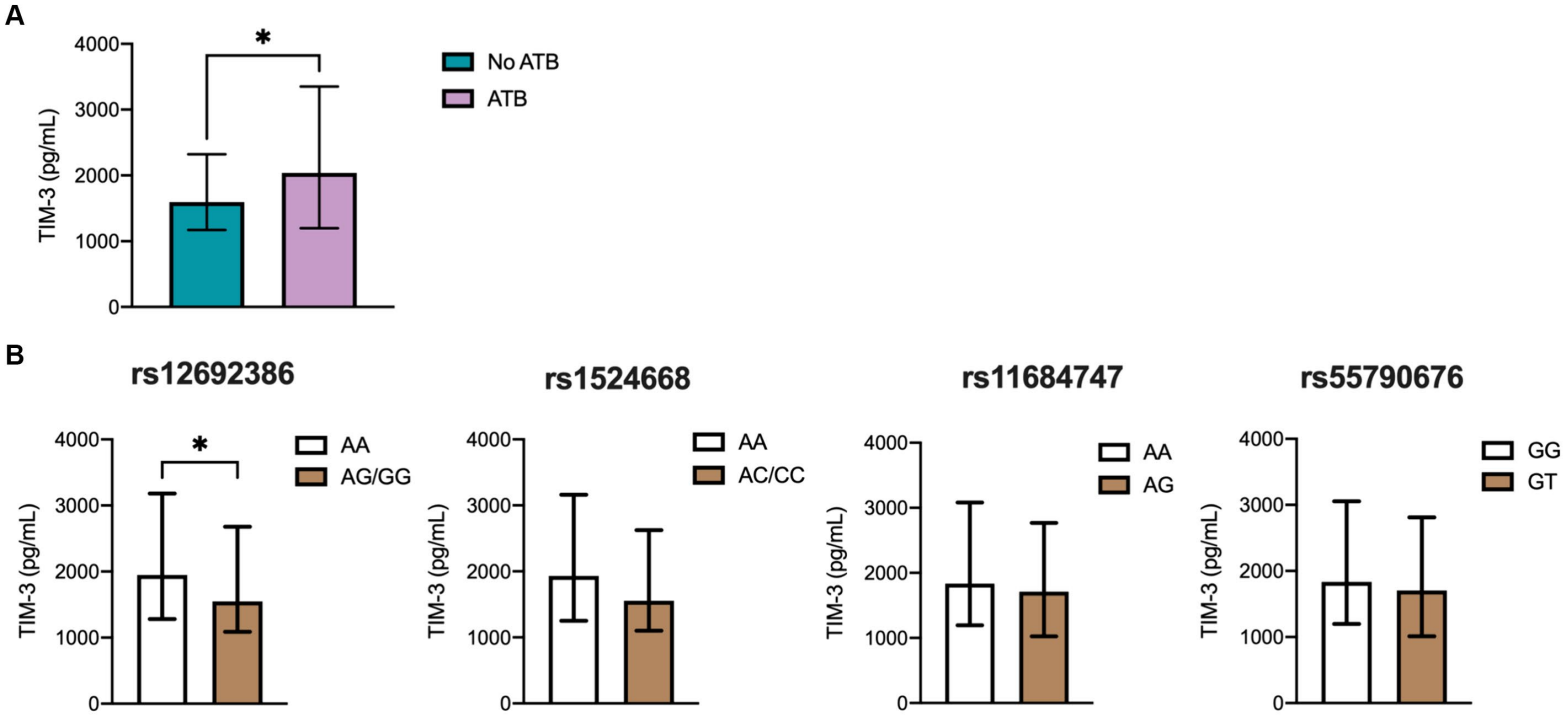
TIM-3 plasma levels in the different TB groups. **(A)** Soluble levels of TIM3 were assessed by ELISA. Genotype distribution according to **(B)** TIM-3 levels in the population, including 224 ATB patients and controls comprehending 70 individuals with LTB (QTF+) and 48 with no LTB (QTF−). Data are displayed as median and IQR values. Differences between groups were performed using the Mann–Whitney U test, *p < 0.05.

T cell immunoglobulin and mucin-domain-3 (TIM-3 or CD366) is a surface molecule expressed by both CD4+ and CD8+ T cells in mice infected with Mtb and in ATB patients (Wang et al., 2011). Although its precise role in TB remains unclear, TIM-3 is cleaved by ADAM17 (Möller-Hackbarth et al., 2013). Together with galectin 9, TIM-3 has been shown to induce antibacterial activity in infected human macrophages (Sada-Ovalle et al., 2012). In this study, we also measured the levels of TIM-3 across various TB patient groups due to the inability to directly measure ADAM17. Nonetheless, if ADAM17 indeed has a significant influence on ATB and cytokine levels, its effect would be reflected in the regulation of other immune molecules besides TNF and TNFRs, such as TIM-3. As illustrated in Figure 3A, ATB patients exhibited significantly higher soluble TIM-3 levels compared to the household control group. Further analysis investigated the association between SNPs of ADAM17 and TIM-3 levels. Notably, individuals homozygous for the AA genotype at rs12692386 had higher systemic TIM-3 levels than those carrying at least one variant allele (Figure 3B). Other molecules cleaved by ADAM17, such as PD-L1 and IL-8, were also evaluated, but their plasma levels were undetectable (data not shown).

### 3.5 Multiple logistic regression analysis reveals an association between ATB status, SNPs of ADAM17, TNF, and TNFRs

A multiple logistic regression analysis was performed, looking for variables associated with ATB status, including SNPs of ADAM17, TNF, and TNFR levels. As observed in Supplementary Table S5, male gender, low weight, tobacco smoking, and diabetes were associated with ATB. Importantly, carrying rs11684747 and rs55790676 SNPs of ADAM17 were inversely correlated with ATB, whereas homozygous to ancestral alleles of these SNPs were positively associated with disease status.

To confirm these findings, we also carried out a multiple regression analysis on rs11684747 and rs55790676 SNPs, considering the TNF, TNFRs, and genotypes. The analytical model was based on the allele with higher frequency in ATB to find a correlation with the disease. This model showed that TNFR1 and TNFR2 correlate with the presence of rs11684747, as well as TNF and TNFR1 individually, TNFR1/TNFR2, and TNF/TNFR1/TNFR2. Moreover, both TNFRs correlate with ATB and AA genotype to rs11684747. The second genotype, homozygous GG to rs55790676 correlated with TNFR1, TNFR2, and both together, but not ATB (Table 4).

**Table 4:** Multiple logistic regression analysis of the association with TB status and levels of TNF and their receptors for the presence of ADAM17 rs11684747 and rs55790676.

Dependent variable	Independent variable	*Z*	*p*-value	OR
rs11684747				
*AA*				
	TB status (ATB)	0.05844	ns	0.7812
	TNF	1.812	0.0701	0.8293
	TNFR1	2.512	**0.0120**	0.9912
	TNFR2	2.682	**0.0073**	0.9967
	TB status (ATB): TNF	1.430	ns	0.6381
	TB status (ATB): TNFR1	1.305	ns	1.004
	TB status (ATB): TNFR2	0.4261	ns	1.001
	TNF: TNFR1	1.998	**0.0457**	1.000
	TNF: TNFR2	1.689	ns	1.000
	TNFR1: TNFR2	2.834	**0.0046**	1.000
	TB status (ATB): TNF: TNFR1	0.9368	ns	1.000
	TB status (ATB): TNF: TNFR2	1.537	ns	1.000
	TB status (ATB): TNFR1: TNFR2	2.035	**0.0419**	1.000
	TNF: TNFR1: TNFR2	2.028	**0.0426**	1.000
rs55790676				
*GG*				
	TB status (ATB)	0.5124	ns	0.1337
	TNF	1.299	ns	0.9050
	TNFR1	2.030	**0.0423**	0.9936
	TNFR2	2.455	**0.0141**	0.9970
	TB status (ATB): TNF	0.9732	ns	0.7794
	TB status (ATB): TNFR1	1.141	ns	1.004
	TB status (ATB): TNFR2	0.9367	ns	1.001
	TNF: TNFR1	1.531	ns	1.000
	TNF: TNFR2	1.532	ns	1.000
	TNFR1: TNFR2	2.463	**0.0138**	1.000
	TB status (ATB): TNF: TNFR1	0.9850	ns	1.000
	TB status (ATB): TNF: TNFR2	0.8013	ns	1.000
	TB status (ATB): TNFR1: TNF	1.891	0.0587	1.000
	TNF: TNFR1: TNFR2	1.669	ns	1.000

Statistical comparisons were performed using Multiple logistic regression with three-way interactions for only the two single nucleotide polymorphisms (SNPs) and alleles frequencies that showed a statistically significant difference between ATB and controls (QFT− and QFT+ individuals). The model included each allele, soluble levels in pg/mL of TNF and TNF receptors, and TB status. The absolute value of *Z*: is calculated as the coefficient estimate divided by its standard error. OR: odds ratio; ns: not significant *p*-value. *p* value in bold show a statistically significant difference.

## 4 Discussion

It is well known that genetic factors play a major role in both infection and clinical progression of TB. Many functional and population-based studies have demonstrated that polymorphisms located in genes of immune responses, as well as in genes related with metabolism, are involved in the susceptibility and the multiplexity of TB-host interactions and pathogenesis (Zuiga et al., 2012). TNF is a pivotal mediator in TB immunopathogenesis (Dorhoi and Kaufmann, 2014). As such, several association animal studies have linked low TNF levels with TB progression and morbidity (Kindler et al., 1989; Flynn et al., 1995; Roach et al., 2002; Mezouar et al., 2019), whereas treatment with TNF blockers is known to result in ATB reactivation and dissemination (Keane et al., 2001; Gómez-Reino et al., 2003; Mohan et al., 2004; Wallis et al., 2004), making TB screening mandatory before anti-TNF therapy (Fernández-Ruiz and Aguado, 2018). The immune effects of TNF are vast and depend on the interactions with either TNFR1 or TNFR2, triggering inflammation, anti-microbial immunity, and cell death. In TB, TNF orchestrates the early induction of chemokines to facilitate leucocyte recruitment and granuloma formation (Roach et al., 2002). Moreover, containment of Mtb infection is attuned to a highly regulated production of this cytokine from different sources, such as myeloid cells and T cells, with several complex mechanisms controlling its abundance and bioavailability to avoid pathogenic functions such as tissue necrosis (Dorhoi and Kaufmann, 2014).

Here, our results showed that TNF levels in ATB patients were lower than in their household contacts. Although previous reports indicated that TB patients have significantly higher TNF levels, those studies used a control group of healthy people with no history of contact with ATB patients, so their immune status against Mtb (QTF or PPD test) is unclear (Mirzaei and Mahmoudi, 2018; Beig et al., 2023). In this sense, our results are in consonance with other studies, where close contacts or healthy controls have higher levels than ATB patients across different age groups (Joshi et al., 2015). Furthermore, TNF expression at the transcriptional level was found to be lower in ATB than in household controls (De Sousa et al., 2023). Our results suggest that TNF might be a readout of loss of protective immunity against Mtb among people exposed to the bacillus, which agrees with a plethora of studies showing that the neutralization of TNF promotes the progression of latent TB infection to ATB (Lin et al., 2010).

One of the processes to optimize TNF expression and function is through the tight control of its interaction with TNFRs. Each of these receptors induces specific immune functions; TNFR1 mediates inflammation and tissue injury (Chavez-Galan et al., 2019), while TNFR2 mediates suppressive activity to reduce excessive inflammation (Chavez-Galan et al., 2017). The transmembrane forms of TNF, TNFR1, and TNFR2 are released into the extracellular space by the protease ADAM17 (Porteu and Nathan, 1990; Black et al., 1997; Mohan et al., 2002; Ruiz et al., 2021), which is essential in establishing a balance between TNF and TNFRs in cell surfaces and tissues. Soluble TNFRs are thought to favor anti-inflammatory functions, acting as decoy receptors of TNF (Kohno et al., 1990). For instance, it has been shown that high TNF levels can induce shedding of TNFR1 by ADAM17, and solTNFR1 then neutralizes circulating TNF, consequently attenuating excessive inflammation (Rowlands et al., 2011).

The metalloprotease function of ADAM17 is not limited to releasing TNF and TNFRs from the cell surface, also shedding more than 80 substrates, including cytokines, growth factors, and cell adhesion molecules receptors, including interleukin (IL)-R1, IL-R6, TGFβ, ACE-2, TIM-3, CD16, among other (Zunke and Rose-John, 2017). Also, it is functionally involved in pathogen recognition and may influence the potential uptake of invading pathogens, inducing the cleavage of PPRs and Toll-like receptors at the macrophages cell surface (Hernandez-Pando et al., 2000). Hence, ADAM17 might be crucial in the immunopathogenesis of several inflammatory disorders and infectious diseases, especially those that depend on TNF signaling and a balanced immune response for protective immunity. Despite this, there is little evidence about the association of ADAM17 with TB, a prototype infectious disease where immune regulation is pivotal in maintaining infection control at the lowest tissue-damage cost during LTB and displaying intense necrosis of granuloma in ATB progressors.

Previous reports suggest that SNPs of ADAM17 are associated with several disorders (Li et al., 2014, 2019; Shao et al., 2016; He et al., 2022; Jung and Hwang, 2022). Importantly, two studies showed that the rs12692386 A > G allele of ADAM17 increased the protease expression and shedding of TNF, IL-6R, and CX3CL1, conferring a higher risk of severe disease and shock in sepsis (Shao et al., 2016; He et al., 2022). Here, we analyzed four SNPs (rs12692386, rs1524668, rs11684747, rs55790676) of ADAM17 looking for a possible association with TB progression. Although we did not find any relationship between rs12692386 A > G and TB susceptibility, we showed that people with rs11684747 A > G and rs55790676 G > T have higher circulating levels of TNF and are overrepresented among healthy close contacts with LTB compared to ATB patients. Together, both studies favor the assumption that ADAM17 takes a role during infection by modulating the production of inflammatory molecules, mainly TNF, which is required for protective immunity during LTB but could be detrimental when it is produced in excess, as in septic shock. In addition, despite rs12692386 being no different between groups, an association of homozygous AA to rs12692386 was found with patients displaying higher TIM-3 systemic levels, suggesting that the presence of this SNP could be involved in inducing an exhausting status in ATB.

In 2000, Hernández-Pando and colleagues demonstrated that the administration of batimastat, a broad-spectrum metalloproteinase inhibitor acting on ADAM17, reduces TB control in mice infected with Mtb via intratracheal instillation (Hernandez-Pando et al., 2000). In these animals, a delay in granuloma formation, higher disease progression, and increased mortality were associated with lower expression of TNF in lung tissue compared to untreated mice. Although these outcomes could be explained by the disruption of other proteases, the data confirm the protective role of TNF in TB, revealing a possible influence of ADAM17 in the process.

In line with this idea, our findings indicate that in healthy close contacts and LTB, ADAM17 acts principally by shedding TNF to maintain concentrations sufficient to keep infection control below a pathological threshold. Much lower levels might make the individual prone to disease progression. Accordingly, we found higher levels of TNF in close contacts than in ATB cases, mainly in those carrying rs11684747 and rs55790676. Thus, the acquisition of certain genetic variants of ADAM17 associated with higher cytokine production might be beneficial. However, in contrast with our results, other reports showed a link between high serum TNF levels and clinical severity of TB measured by chest X-ray alterations, weight loss, and positive PPD skin test (Júnior et al., 2008; Beig et al., 2023), whereas Kart et al. (2003) found no relationship between increased TNF levels and TB outcomes. Unfortunately, we had no clinical data on the severity of the disease in our ATB cohort. Hence, looking for differences in ATB outcomes according to TNF levels and ADAM17 SNPs is warranted for future investigations.

On the other hand, our data suggest a little effect of ADAM17 on TNFRs during LTB or resistance to infection status since the levels of these receptors were lower in healthy close contacts of ATB cases. Also, we did not find a relationship between SNPs and TNFR1 and TNFR2, although TNFRs levels tended to be lower in people carrying the rs11684747 A > G and rs55790676 G > T SNPs of ADAM17. Of note, the levels of TNFRs, mainly TNFR2, were higher in ATB patients compared to controls. Interestingly, a study in mice infected with aerosolized Mtb found high levels of TNFRs, especially TNFR2, in bronchioalveolar lavage specimens collected one month after inoculation. In this model, the investigators discovered that mannose-capped lipoarabinomannan (ManLAM) was sufficient to promote ADAM17 shedding of TNFR2, and a lower level of TNFR1, hindering the release of tmTNF (Richmond et al., 2012). In agreement with our findings, they also showed higher levels of TNFR2 among ATB patients.

Therefore, we believe that in close contacts resistant to Mtb infection and those with LTB, ADAM17 plays a protective role in keeping TNF levels in adequate concentrations. Nonetheless, when protective immunity is disrupted by age, diabetes, or immune suppression, proliferation of bacilli increases the amount of ManLAM and other virulence factors, consequently promoting TNFR2 and TNFR1 release, which in turn block TNF signaling, further hindering protective immunity against Mtb. In this process, certain SNPs of ADAM17 might confer protection or susceptibility if they enhance the ability to maintain a favorable balance between TNF and TNFRs. This might extend to other infections like COVID-19, where a disbalance of proinflammatory and anti-inflammatory signals is crucial to determine clinical outcomes. Interestingly, the rs55790676 polymorphism was recently associated with an increased susceptibility to COVID-19 in India (Patil et al., 2021). Also, it is possible that this polymorphism might influence the expression of entry factors important for SARS-CoV-2 infection, as demonstrated by functional studies showing that a lower expression of ADAM17 provokes a reduced release of ACE2 from the cell membrane (Healy and Lilic, 2021), whereas increased protease activity primes the viral S protein favoring susceptibility to SARS-CoV-2 infection (Jocher et al., 2022).

The current study has limitations that must be considered when interpreting the results. Firstly, the small sample size limited our statistical power to examine a small genetic effect, decreasing the accuracy of our genotype–phenotype analyses and hindering the possibility of making more comparisons between certain groups of participants. Due to low allele frequency, we could not identify enough individuals homozygous GG to rs11684747 and TT to rs55790676. Also, the case–control design of this study may bias any true relationship between ADAM17 SNPs and ATB. In addition, the rs11689958 allele was not assessed in our study, which shows a solid pairwise linkage disequilibrium with other SNPs at the ADAM17 promoter region (Shao et al., 2016). Finally, due to technical limitations, we were unable to directly measure ADAM17 in our study. However, we hypothesized that if this protease significantly influences cytokine levels during TB, its effect would be reflected in the regulation of other immune molecules cleaved by ADAM17 that are involved in disease immunopathogenesis. Consequently, we chose to measure TIM-3 levels. TIM-3 is a co-inhibitory molecule that regulates T cell function and may play a role in TB. Recent research indicates that TIM-3 acts as an exhaustion marker in CD4+ and CD8+ T cells during Mtb infection (Jayaraman et al., 2016). Interestingly, we found higher levels of TIM-3 in ATB patients and those homozygous to the rs12692386 SNP of ADAM17, suggesting its involvement in chronic infection, which is in line with previous reports (Chen et al., 2022).

Hence, despite these caveats, our results provide preliminary data that open a novel and unexplored hypothesis of a possible role for ADAM17 in TB; however, the role of these SNPs in latent TB is unknown and requires further investigation in future larger studies. In sum, our main findings were that levels of TNF and TNFRs are influenced by SNPs of ADAM17 and distinguish ATB patients from close contacts. We observed that homozygous AA to rs11684747 and GG to rs55790676 exhibited lower plasma levels of TNF than heterozygous individuals. In contrast, homozygous patients AA to rs11684747 and GG to rs55790676 showed lower levels of TNFR2 when compared to heterozygous individuals. Multiple regression analyses showed a significant association between the levels of TNF, TNFR1, TNFR2, tuberculosis status, and the AA homozygosity on the rs11684747 SNP. This study suggests a role for SNPs of ADAM17 in genetic susceptibility to ATB that warrants further research.

## Data availability statement

The datasets presented in this study can be found in online repositories. The names of the repository/repositories and accession number(s) can be found below: https://www.ncbi.nlm.nih.gov/clinvar/, SCV005043058, SCV005043059, SCV005043068, SCV005043069.

## Ethics statement

The studies involving humans were approved by the study was approved by the Institutional Review Board of Instituto Nacional de Enfermedades Respiratorias Ismael Cosío Villegas (protocol code B04-23, approved in February 2023). Informed consent was obtained from all subjects involved in the study. The studies were conducted in accordance with the local legislation and institutional requirements. The participants provided their written informed consent to participate in this study.

## Author contributions

JC-P: Conceptualization, Methodology, Writing – original draft, Formal analysis, Investigation. LR-L: Conceptualization, Formal analysis, Investigation, Methodology, Writing – original draft, Supervision. MC: Methodology, Writing – review & editing. EO-M: Methodology, Writing – review & editing. AT-G: Methodology, Writing – review & editing. MM-M: Methodology, Writing – review & editing. AC-L: Methodology, Writing – review & editing, Validation. GR-M: Methodology, Writing – review & editing. IG-G: Methodology, Writing – review & editing. JR-N: Writing – review & editing, Investigation. BG-P: Methodology, Writing – review & editing. KL-S: Methodology, Writing – review & editing. FJ-J: Methodology, Writing – review & editing. PG-O: Methodology, Writing – review & editing. CS-L: Methodology, Writing – review & editing. KB-P: Formal analysis, Writing – review & editing. MM-T: Data curation, Writing – review & editing. LC-G: Conceptualization, Methodology, Resources, Supervision, Writing – original draft, Writing – review & editing. JZ: Data curation, Formal analysis, Funding acquisition, Methodology, Project administration, Writing – original draft, Writing – review & editing.
