# Supplementary material for: The rs11684747 and rs55790676 SNPs of ADAM17 influence tuberculosis susceptibility and plasma levels of TNF, TNFR1, and TNFR2
Source: Front Microbiol. 2024 May 31;15:1392782. doi: 10.3389/fmicb.2024.1392782 (PMC11177089; doi:10.3389/fmicb.2024.1392782)
Supplement: Supplementary file 1 [file Data_Sheet_1.pdf]

Supplementary Material

Supplementary Table S1. Demographic and clinical characteristics of patients in the different TB groups.

Characteristics	Overall N = 342	Group			
		QTF- N = 48	QTF+ N = 70	ATB N = 224	p-value
Male gender	138 (40%)	14 (29%)	16 (23%)	108 (48%)	<0.001
Age	43.5 (31.0, 56.0)	38.5 (28.0, 54.0)	44.0 (32.2, 53.8)	43.5 (31.0, 57.0)	0.5
BMI	23.3 (20.1, 27.2)	27.8 (24.8, 30.0)	29.2 (25.3, 32.9)	21.3 (18.9, 24.4)	<0.001
Obesity	48 (14%)	12 (25%)	26 (37%)	10 (4.5%)	<0.001
Low weight	79 (23%)	0 (0%)	2 (2.9%)	77 (34%)	<0.001
Smoking	85 (36%)	10 (21%)	18 (26%)	57 (47%)	<0.001
Diabetes	95 (28%)	2 (4.2%)	9 (13%)	84 (38%)	<0.001
SAH	49 (14%)	6 (12%)	8 (11%)	35 (16%)	0.6
BCG	249 (88%)	44 (92%)	61 (87%)	144 (87%)	0.7

Data are shown as n (%) or median (IQR). Differences between groups were analyzed using Pearson's Chi-squared test, Kruskal-Wallis rank sum test, or Fisher's exact test, as appropriate. ATB, active pulmonary tuberculosis; BCG, Bacillus Calmette-Guerin vaccine; BMI, body mass index; QTF, QuantiFERON test; SAH, systemic arterial hypertension; TB, tuberculosis.

Supplementary Table S2. Genotypes and allele frequencies in the different TB groups.

Characteristics	Overall N = 342	Group			
		QTF- N = 48	QTF+ N = 70	ATB N = 224	p-value
rs12692386					
AA	206 (61%)	31 (65%)	37 (53%)	138 (62%)	0.3
AG	112 (33%)	16 (33%)	27 (39%)	69 (31%)	0.5
GG	21 (6.2%)	1 (2.1%)	6 (8.6%)	14 (6.3%)	0.4
A	318 (94%)	47 (98%)	64 (91%)	207 (94%)	0.4
G	133 (39%)	17 (35%)	33 (47%)	83 (38%)	0.3
rs1524668					
AA	222 (65%)	31 (65%)	40 (57%)	151 (68%)	0.2
AC	100 (29%)	16 (33%)	26 (37%)	58 (26%)	0.2
CC	18 (5.3%)	1 (2.1%)	4 (5.7%)	13 (5.9%)	0.7
A	322 (95%)	47 (98%)	66 (94%)	209 (94%)	0.7
C	118 (35%)	17 (35%)	30 (43%)	71 (32%)	0.2
rs11684747					
AA	293 (86%)	41 (85%)	54 (77%)	198 (89%)	0.039
AG	47 (14%)	7 (15%)	16 (23%)	24 (11%)	0.039
GG	0 (0%)	0 (0%)	0 (0%)	0 (0%)	

A	340 (100%)	48 (100%)	70 (100%)	222 (100%)	
G	47 (14%)	7 (15%)	16 (23%)	24 (11%)	0.039
rs55790676					
GG	297 (87%)	41 (85%)	55 (79%)	201 (90%)	0.039
GT	44 (13%)	7 (15%)	15 (21%)	22 (9.9%)	0.039
TT	0 (0%)	0 (0%)	0 (0%)	0 (0%)	
G	341 (100%)	48 (100%)	70 (100%)	223 (100%)	
T	44 (13%)	7 (15%)	15 (21%)	22 (9.9%)	0.039

Data are displayed as n (%). Differences between groups were analyzed using the Pearson's Chi-squared test or Fisher's exact test, as appropriate.

Supplementary Table S3. TNF and TNF receptors plasma levels in ATB and control groups.

**Characteristics**	**Overall N = 342**	**Disease progression**		**p-value**
		**ATB N = 224**	**No ATB N = 118**	
TNF	11.7 (7.8, 17.6)	10.1 (7.1, 14.6)	14.4 (8.9, 24.4)	<0.001
TNFR1	1,009.0 (755.5, 1,509.1)	1,261.5 (963.9, 1,800.9)	811.1 (652.4, 1,010.3)	<0.001
TNFR2	3,252.4 (2,420.8, 4,089.8)	3,739.4 (2,693.0, 4,765.4)	2,706.0 (1,952.9, 3,486.0)	<0.001

Data are displayed as median (IQR). Differences between groups were analyzed using the Wilcoxon rank sum test.

Supplementary Table S4. TNF and TNF receptors plasma levels in the different TB groups.

**Characteristics**	**Overall N = 342**	**Group**			**p-value**
		**QTF- N = 48**	**QTF+ N = 70**	**ATB N = 224**	
TNF (pg/mL)	11.7 (7.8, 17.6)	12.3 (8.0, 21.6)	15.6 (10.7, 36.4)	10.1 (7.1, 14.6)	<0.001
TNFR1 (pg/mL)	1,009.0 (755.5, 1,509.1)	803.2 (618.1, 993.0)	821.1 (677.9, 1,026.6)	1,261.5 (963.9, 1,800.9)	<0.001
TNFR2 (pg/mL)	3,252.4 (2,420.8, 4,089.8)	2,775.2 (2,223.9, 3,439.4)	2,622.2 (1,873.0, 3,501.5)	3,739.4 (2,693.0, 4,765.4)	<0.001

Data are displayed as median (IQR). Differences between groups were analyzed using the Kruskal-Wallis with post-hoc Dunn´s test.

Supplementary Table S5. Multiple regression analysis of the variables associated with ATB progression.

**Characteristic**	OR	95% CI	p-value	Adjusted p-value[1]
Male gender	2.73	1.69, 4.51	<0.001	<0.001
Age	1.01	0.99, 1.02	0.345	0.405
BMI	0.78	0.73, 0.82	<0.001	<0.001
Obesity	0.10	0.04, 0.20	<0.001	<0.001
Low weight	30.4	9.29, 187	<0.001	<0.001
Smoking	2.86	1.66, 5.03	<0.001	<0.001
Diabetes	5.84	3.08, 12.1	<0.001	<0.001
SAH	1.38	0.72, 2.75	0.339	0.405
BCG	0.85	0.40, 1.75	0.661	0.661
rs12692386				
AA	1.22	0.77, 1.93	0.388	0.408
AG/GG	0.82	0.52, 1.29	0.388	0.408
rs1524668				
AA	1.41	0.88, 2.24	0.150	0.200
AC/CC	0.71	0.45, 1.13	0.150	0.200
rs11684747				
AA	2.00	1.07, 3.73	0.030	0.047
AG/G	0.50	0.27, 0.94	0.030	0.047
rs55790676				
GG	2.09	1.10, 3.98	0.024	0.044
GT/T	0.48	0.25, 0.91	0.024	0.044
Cytokine levels				
TNF	0.93	0.90, 0.96	<0.001	<0.001
TNFR1	1.00	1.00, 1.00	<0.001	<0.001
TNFR2	1.00	1.00, 1.00	<0.001	<0.001

OR, Odds Ratio; CI, Confidence Interval. 1 False discovery rate correction for multiple testing.

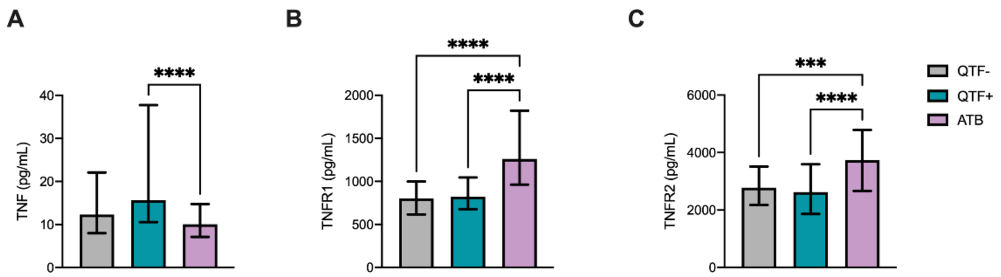

Supplementary Figure S1. TNF and TNF receptors plasma levels in the different TB groups. Soluble levels of TNF (A), TNFR1 (B), and TNFR2 (C) were assessed by ELISA in QTF- (n=48), QTF+ (n=70), and ATB (n=224) patients. Data are displayed as median and IQR values. Differences between groups were analyzed using the Kruskal-Wallis with post-hoc Dunn´s test, ***p < 0.001, and **** p < 0.0001.
